# Regurgitant leak from the area between the stent post and the sewing ring of a stented bovine pericardial valve implanted in the aortic valve position

**DOI:** 10.1186/1476-7120-8-52

**Published:** 2010-11-28

**Authors:** Masataka Kuroda, Takashi Sudo, Shiro Koizuka, Koichi Nishikawa, Yuji Kadoi, Shigeru Saito

**Affiliations:** 1 Department of Anesthesiology, Gunma University Graduate School of Medicine, 3-39-22 Showa-machi, Maebashi City, Gunma 371-8511, Japan

## Abstract

Biologic valves can sometimes have a small closure or leakage backflow jet originating from the central coaptation point. This is physiologic regurgitation that usually only requires monitoring, and not treatment.

Another non-central transvalvular leakage is occasionally seen in both porcine and pericardial valves and originates from the base of the stent post. Typically, it spontaneously decreases or even disappears by the end of the surgery, after administration of protamine. This leak, however, needs to be distinguished from abnormal paravalvular leakages, especially if the regurgitation is relatively large, as this may require an extra cardio-pulmonary bypass (CPB) run.

In our case with stented bovine pericardial valves, detailed transesophageal echocardiography (TEE) examination immediately after CPB showed oblique and turbulent flow, which originated from the base of the stent post and flowed toward the anterior mitral leaflet. An extra CPB run, assessment of the cause of the leakage, and restoration if necessary, might have been required if the leakage did not improve or was exacerbated, because contact of the anterior mitral valve leaflet by the oblique flow is associated with the risks of infective endocarditis and hemolysis. Detailed TEE examination accurately delineated the site of the leak, which was subsequently found to originate from the site between the anterior stent post and the sewing ring. The leakage in this case was classified as non-paravalvular, non-central leakage within the sewing ring. Accurate diagnosis of the leakage by intra-operative TEE led to the decision to administer protamine and to adopt a wait-and-watch approach.

## Background

Biologic valves can sometimes have a small closure or leakage backflow jet originating from the central coaptation point [1]. This is physiologic regurgitation that usually only requires monitoring, and not treatment. Another non-central type of leakage within the sewing ring is also known, though only a few cases have been reported [2,3]. The leak, which originates from the fabric-covered regions of the stent struts or from the region between the stent and the sewing ring in stented porcine heterografts or stented bovine pericardial valves [1-3], is not classified as a paravalvular leak because it originates inside the sewing ring. Instead, it is classified as non-central transvalvular leakage which originates outside the bioprosthetic annulus [2,3]. The leak might be due to a structural problem associated with the material of the fabric, because these regurgitant jets leak through the fabric of the bioprosthetic valve [1].

Non-central transvalvular leakage is occasionally seen in both porcine and pericardial valves and originates from the base of the stent post. Typically, it spontaneously decreases or even disappears by the end of the surgery, after administration of protamine, or resolves over time as the fabric becomes sealed with cellular elements or endothelium [1,2]. It seems to be unlike signature flow patterns, and is usually trivial or mild. This leak, however, needs to be distinguished from abnormal paravalvular leakages, especially if the regurgitation is relatively large, as this may require an extra CPB run.

## Case presentation

A 59-year-old man (weight, 76 kg; height, 163 cm) with a history of hypertension and smoking was scheduled for aortic valve replacement (AVR) to treat severe aortic stenosis due to a bicuspid aortic valve. Echocardiography revealed an aortic valve area of 0.5 cm^2 ^and a mean pressure gradient of 51 mmHg across the stenotic aortic valve. Left ventricular hypertrophy was evident with normal systolic function and grade 1 diastolic dysfunction.

After anesthetic induction and tracheal intubation, a 5-MHz multiplane TEE probe (Agilent Technologies, Andover, MA) was inserted into the esophagus. An initial, intraoperative, pre-CPB TEE revealed severe aortic valve stenosis due to a bicuspid aortic valve. The valve was replaced with a 21-mm Carpentier-Edwards Perimount Magna aortic valve, model 3000 (Edwards Lifesciences, Irvine, CA, USA). The patient was weaned off CPB on low-dose dopamine and dobutamine.

**Figure 1:**
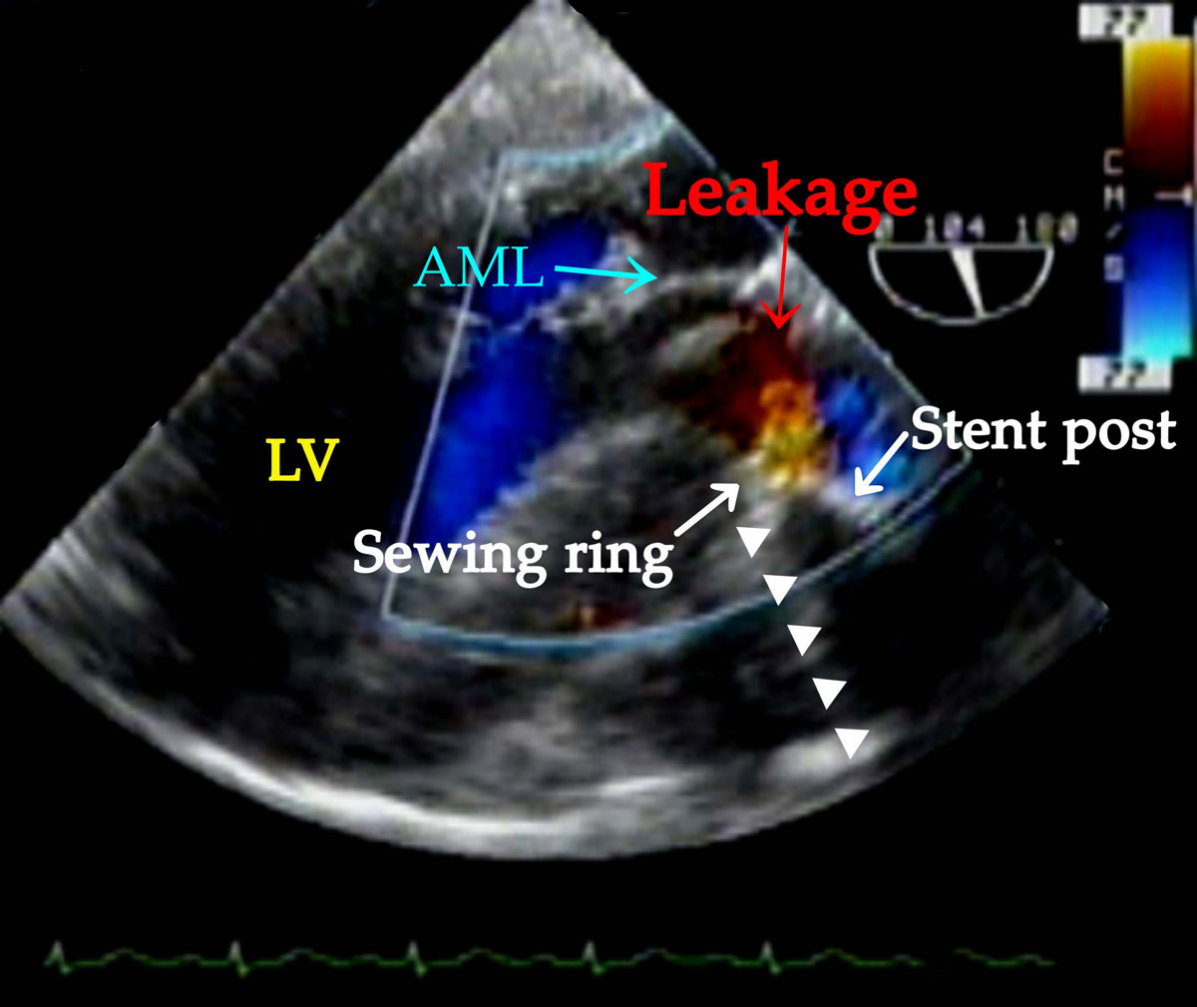
**Transesophageal echocardiography showing leakage between the stent-post and the sewing ring before administration of protamine**. Trans-gastric long axis view shows the diastolic oblique regurgitant flow toward the anterior mitral leaflet (red arrow) between the stent-post (white arrow) and the sewing ring (white arrow) before administration of protamine and immediately after CPB. The acoustic shadow (triangle) is accompanied by the stent-post and the sewing ring. LV: left ventricle; AML: anterior mitral leaflet.

**Figure 2:**
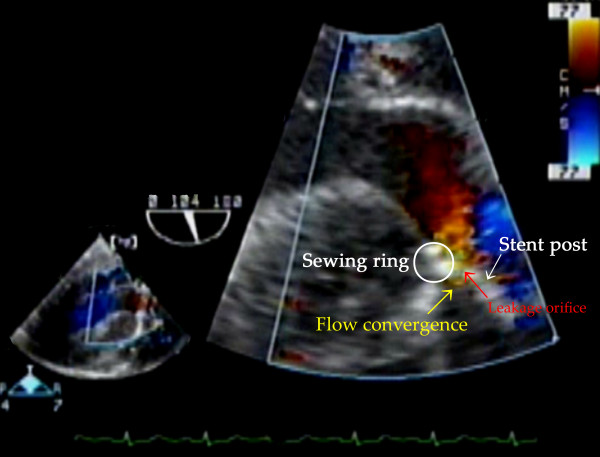
**Zooming in on the leakage site**. A little flow convergence (yellow arrow) was seen at the root of the leakage between the stent-post and the sewing ring. The possible site of the leakage is indicated by the red arrow.

**Figure 3:**
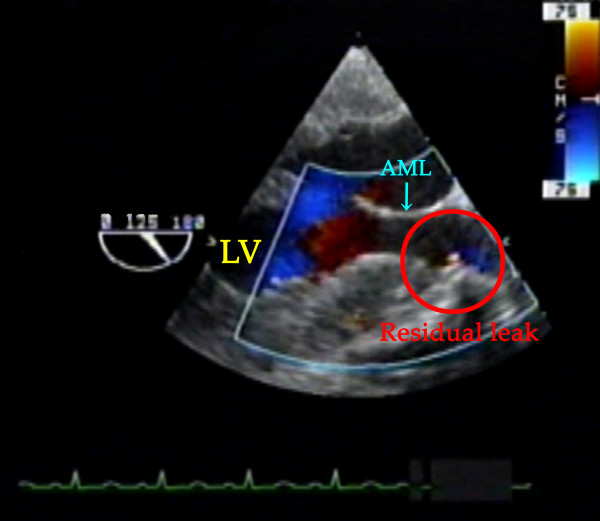
**Transesophageal echocardiography showing leakage between the stent-post and the sewing ring at the end of surgery**. At the end of the operation, about 90 min after protamine administration, the residual leak (red circle), as observed by the zoomed TG LAX view, had almost disappeared.

TEE examination immediately after CPB confirmed a well seated AV prosthesis with normal leaflet mobility in the mid esophageal (ME) AV short-axis (SAX), ME AV long-axis (LAX) and trans-gastric (TG) LAX views. Further examination with color flow Doppler (CFD) showed turbulent flow from outside to inside at the region corresponding to the anterior stent-post in the ME SAX view with the multiplane angle at 30° and a field depth of 10 cm, originating from outside of the stent-post (Additional file 1, Video clip 1). The regurgitant flow was revealed as being between the stent-post and the sewing ring in the TG LAX view with the multiplane angle at 104°, a field depth of 10 cm, and velocity range of 77 cm/sec (Figure 1) (Additional file 1, Video clip 1). On zooming in on the region, a little flow convergence was seen at the root of the leakage between the stent-post and the sewing ring (Figure 2) (Additional file 1, Video clip 1). This was identified as the possible site of the leak orifice. This flow profile was consistent with oblique regurgitation throughout diastole, which flowed from anterior to posterior, toward the anterior mitral leaflet. Twenty minutes after separation from CPB, protamine was administered, following which the regurgitation decreased considerably (Additional file 2, Video clip 2). At the end of the operation, about 90 min after protamine administration, regurgitation had almost disappeared in the TG LAX view with the multiplane angle at 125°, a field depth of 10 cm and velocity range of 75 cm/sec (Figure 3) (Additional file 2, video clip 2). Throughout the procedure, when assessing leakages with CFD, the velocity range in the color scale was set to the highest possible level to avoid overestimation of the regurgitation.

The patient spent 5 days in the intensive care unit and was discharged from the hospital 17 days later. Post-operative transthoracic echocardiography 11 days after the operation confirmed the absence of regurgitation.

## Discussion

Intraoperative TEE can accurately diagnose the site of a valvular leakage. Diagnosing the site of a leakage of the mitral valve is relatively easy because of the appropriate angle of the acoustic beam relative to the mitral valve and the surrounding fields. On the other hand, diagnosis of aortic valve regurgitation by TEE is relatively difficult because of the angle of the acoustic beam and aortic valve field, especially in the ME view. Since the acoustic beam interrogates laterally to the bio-prosthetic aortic valve in the ME view, observation of the anterior side of the valve is particularly affected by the acoustic shadow, which is produced by the proximal side of the sewing ring. This can be overcome by use of the TG-LAX view, which is effective in observing the anterior side of the aortic valve without the effect of the acoustic shadow, because the acoustic beam interrogates from the left ventricular outflow tract side. Furthermore, the ME-SAX view is also effective in indicating the location of the leak. Slight advancement of the TEE probe from the view at the level of the valvular tip enables observation of the entire base of the stent post where leakage through the fabric-covered region originates, without the effects of acoustic shadowing. Therefore, diagnosis of the site of the leakage can be well done with a combination of ME-SAX and TG-LAX views.

In our case with the stented bovine pericardial valve, leakage at the site of the anterior stent post was initially observed by slightly advancing the TEE probe from the ME-AV-SAX view. The leakage was seen to originate from outside the stent post, although the border of the origin of the leakage was not clear. Then, the TG-LAX view was applied to accurately delineate the site of the leak, which was subsequently found to originate from the site between the anterior stent post and the sewing ring. Hence, the leakage in this case was classified as non-paravalvular, non-central leakage within the sewing ring, which led to the decision to administer protamine and to adopt a wait-and-watch approach. An extra CPB run, assessment of the cause of the leakage, and restoration if necessary, however, might have been needed if the leakage did not improve or was exacerbated, because contact of the anterior mitral valve leaflet by the oblique flow is associated with the risks of infective endocarditis and hemolysis. Recently, we experienced a similar leakage after AVR with the same type of valve. A 67-year-old man (weight, 66 kg; height, 170 cm) with a history of hypertension and mild anemia was scheduled for aortic valve replacement (AVR) to treat severe aortic regurgitation. Echocardiography revealed severe aortic regurgitation and a dilated left ventricle with impaired systolic function, as indicated by an ejection fraction of 47%. The valve was replaced with a 21-mm Carpentier-Edwards Perimount Magna aortic valve, model 3000 (Edwards Lifesciences, Irvine, CA, USA). TEE examination with color flow Doppler (CFD) showed turbulent flow from outside to inside at the region corresponding to the anterior stent-post in the ME SAX and TG LAX views. This flow profile was consistent with oblique regurgitation throughout diastole, which flowed from anterior to posterior, toward the anterior mitral leaflet. In this other case, the cause of the leakage was evaluated during the extra CPB run and cardiac arrest, although no abnormality was detected in the valve and its surroundings. Once again, the leak disappeared by the end of the surgery, after administration of protamine. This previous experience aided the decision to conservatively manage the current case.

**Figure 4:**
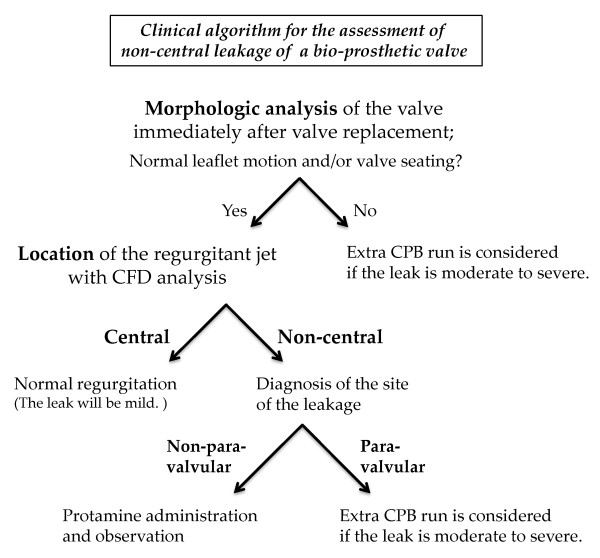
**Clinical algorithm for assessment of non-central leakage of a bio-prosthetic valve**. After assessment of the morphology of the implanted valve demonstrating leaflet motion and valve seating, the location of the leak, whether central or non-central, is assessed by CFD. If the leak is non-central, further detailed examination is needed to determine the location of the leak and whether it is non-paravalvular or paravalvular. If the leak is non-paravalvular, it is possible that the leak is the same as in this case and would require only protamine administration and observation.

When confronted with unusual regurgitation after valve replacement, as in these cases, the leakage should be assessed to determine whether it is normal or pathological. The morphology of the valve and the location of the leak are important knowledge for the assessment. A clinical algorithm for the general assessment of non-central leakage of a bio-prosthetic valve is shown in Figure 4. Evaluation of the severity of leakages from prosthetic valves is challenging due to the limitations of ultrasonography. Although quantitative analysis is more difficult, clinical decisions about management of the leaks are usually based on the presence of pathologic prosthetic regurgitation and its clinical consequences, such as hemolysis, and heart failure, and not on exact measurements of the severity. Three-dimensional analysis will be able to assist in the assessment of the morphology of the valve and the location of the leak.

## Conclusion

Detailed TEE examination with a combination of ME-SAX and TG-LAX views enables diagnosis of the exact location of the leakage of stented bovine pericardial valves implanted in the aortic valvular position. Exact diagnosis of the leakage with intra-operative TEE is necessary to be able to appropriately deal with the condition.

## Consent

Written informed consent was obtained from the patient for publication of this case report and any accompanying images. A copy of the written consent is available for review by the Editor-in-Chief of this journal.

## Abbreviations

(AVR): Aortic valve replacement; (CPB): cardio-pulmonary bypass; (CFD): color flow Doppler; (LAX): long-axis; (ME): mid esophageal; (SAX): short-axis; (TEE): transesophageal echocardiography; (TG): trans-gastric;

## Competing interests

The authors declare that they have no competing interests.

## Authors' contributions

MK conceived the case report, performed intra-operative echocardiographic examinations, reviewed literature and wrote the manuscript. TS and SK has been involved in drafting the manuscript. KN and YK contributed to the critical revision of the manuscript. SS supervised and commented on the manuscript. All authors read and approved the final manuscript.

## Supplementary Material

Additional file 1 **The regurgitation before administration of protamine**. Before administration of protamine, examination with color flow Doppler (CFD) showed turbulent flow (red arrow) from outside to inside at the region corresponding to the anterior stent-post in the ME AV SAX view, originating from outside the stent-post. A diastolic oblique regurgitant flow (red arrow) was revealed between the stent-post and the sewing ring in the TG LAX view. This flow profile was consistent with oblique regurgitation, which flowed from anterior to posterior, toward the anterior mitral leaflet. LA: left atrium; LV: left ventricle. Click here for file

Additional file 2 **The regurgitation after administration of protamine**. The regurgitation decreased considerably after protamine administration (yellow circle). At the end of the operation, about 90 min after protamine administration, regurgitation in the TG LAX view had almost disappeared (yellow circle). Click here for file
